# Survival of Patients with Small Cell Lung Cancer in King Abdulaziz University Hospital, Jeddah, Saudi Arabia

**DOI:** 10.7759/cureus.6648

**Published:** 2020-01-13

**Authors:** Faris Alhejaili, Marwan Al-Hajeili, Salwa I Bakhsh, Nujood S Banjar, Wejdan Alghamdi, Atheer F Alsulami, Maha Algamdi

**Affiliations:** 1 Department of Medicine, King Abdulaziz University, Jeddah, SAU; 2 Department of Pathology, King Abdulaziz University, Jeddah, SAU

**Keywords:** lung cancer, small cell lung cancer, sclc, saudi arabia, king abdulaziz university

## Abstract

Background

Lung cancer is estimated to be 12% of all new cases of cancer. It is one of the most common cancers in men and women, and it is the main cause of cancer-related death in the United States of America. More than 90% of small cell lung cancer (SCLC) patients are elderly, with a current or past history of smoking. In Saudi Arabia, lung cancer incidence is low as compared to the global incidence. In 2013, the age-standardized ratio (ASR) was 1.8 per 100,000 for females and 5.5 per 100,000 for males. In our study, we aimed to assess the outcomes of SCLC at King Abdulaziz University Hospital (KAUH), Jeddah, Saudi Arabia.

Methods

This retrospective cohort study included all patients aged 14 years and older with a diagnosis of SCLC from 2007 to 2017 using electronic medical records at KAUH.

Data analysis was performed using Stata SE, version 15.0 (StataCorp LLC, TX). The primary outcome of this study was the survival of patients diagnosed with SCLC. Survival was defined as the time the patient lived in months from the date of pathological diagnosis to the date of the last follow-up or death. We included all variables in a univariate and multivariate analysis to determine the hazard ratio for each variable.

Results

In our study, we initially collected 193 lung cancer cases diagnosed during the period of 2007 to 2017 at KAUH, which was then narrowed to 22 after the selection of only SCLC cases.

Data obtained showed 20 males (90.91%) and two females (9.09%), the median age of diagnosis was 64 years, and 45% of patients are active smokers, 9% are ex-smokers, and the smoking status of 41% of patients is unknown. Our data showed an overall median survival of 6.4 months (interval=11).

Conclusion

We observed that more than half of our patients who received chemotherapy showed improvement and a higher survival rate than those who didn't. In addition, 19% who received radiation therapy showed improvement and a higher survival rate than those who didn't. Future efforts to address the major issues that surround SCLC survivors, and to formulate a comprehensive survivorship care plan are required to develop better outcomes in survival and to improve the overall quality of life to pretreatment levels.

## Introduction

Lung cancer, one of the most common cancers in men and women and the main cause of cancer-related death in the United States, is estimated to make up 12% of all new cases of cancer [1]. In 2008, more than 1.6 million lung cancer cases were newly diagnosed worldwide, with nearly 1,378,400 deaths caused by the disease [2]. The age-standardized ratio (ASR) globally in 2008 was 33.8 per 100,000 for males and 13.5 per 100,000 for females [1]. In contrast, in Saudi Arabia, the incidence of lung cancer is comparatively low; in 2013, for example, it was 5.5 per 100,000 for males and 1.8 per 100,000 for females [3].

The incidence of all types of lung cancer has been declining in the United States with the onset of tobacco smoking cessation programs [2]. Moreover, with the advent of low-dose spiral computed tomography (CT) scanning for the early detection of lung cancer, overall survival rates for lung cancer patients have increased by about 5%. Nonetheless, the overall five-year survival rates remain dismal at only 6% [1-2,4].

Small cell lung cancer (SCLC) accounts for 13% of all newly diagnosed cases of lung cancer worldwide [5], with more than 180,000 patients diagnosed per year [6]. It is an aggressive neuroendocrine malignancy characterized by a high growth fraction, short doubling time, and the early development of widespread metastases [7]. More than 90% of patients with SCLC are older adults with a history of smoking. Tobacco exposure has been shown to be strongly associated with the development of SCLC, and the duration and intensity of smoking increase the risk [6]. Despite the fact that it is a chemotherapy- and radiation therapy-sensitive disease, SCLC typically recurs rapidly following primary treatment and only 6% of patients are alive five years after diagnosis [1].

With the population of Saudi Arabia growing, including that of older adults, and the prevalence of smoking high and gradually rising, increasing by 1.5% for males and 2.0% for females, the incidence of lung cancer also has the potential to increase. In this study, we, therefore, aimed to evaluate the outcomes among patients 14 years and older who were diagnosed with SCLC at King Abdulaziz University Hospital (KAUH), Jeddah, Saudi Arabia, from 2007 to 2017.

## Materials and methods

This retrospective cohort study was performed at KAUH, Jeddah, Saudi Arabia. The study was approved by the research ethics committee at the Faculty of Medicine at KAUH. It involved sampling and analyzing all patients age 14 years and above with a diagnosis of SCLC at KAUH.

Medical records from 2007 to 2017 were collected and reviewed. On the basis of our pathology review, of a total of 193 patients with lung cancer, we selected 22 who were diagnosed with SCLC. The collected samples were analyzed for demographic data, date of diagnosis, type of cancer (primary or secondary), histological type (adenocarcinoma, squamous cell carcinoma, large cell carcinoma, small cell carcinoma, non-SCLC (NSCLC), carcinoid, and unspecified), cytological type (negative, positive, atypical, and insufficient), type of biopsy, surgical treatment, margin involvement, lymph node involvement, chemotherapy, radiation therapy, date of last follow-up, and date of death.

The data of all patients in our study were collected from the electronic medical records of KAUH (Phoenix) except for patients who were diagnosed in 2007 and 2008. Because Phoenix was established in 2009, for the two earliest years, we collected hard copies of the patients’ pathology reports from the Pathology department. The data were revised and reviewed thoroughly by the main investigators to prevent misinterpretation.

Data analysis was performed by using Stata SE version 15.0 (StataCorp LLC, TX). The main outcome of this study was the survival rate of patients diagnosed with SCLC. Survival was interpreted as the period in months during which the patient was alive from the date of the pathological diagnosis to the date of death or the last follow-up. We incorporated all variables in a univariate and multivariate analysis to establish the hazard ratio for each variable.

## Results

Demographic characteristics

Data obtained for the 22 SCLC cases showed that there were 20 males (90.91%) and two females (9.09%) and that the median age of diagnosis was 64 years. Among these patients, 45% were active smokers, 9% were ex-smokers, and 41% had unknown smoking status. Thirteen patients (59.09%) received chemotherapy and four (18.18%) received radiotherapy, but none of the patients (0%) had a surgical intervention (Table 1).

**Table 1:** Characteristics of patients treated for SCLC SCLC: small cell lung cancer ^a ^Shown as number of cases except where otherwise indicated

Characteristic	Value	Cases^a ^ (N = 22)	Percentage
Age, years, mean		64.57	
Gender	1 Male	20	90.91
	2 Female	2	9.09
Nationality	1 Saudi	7	31.82
	2 Non-Saudi	15	68.18
Cytological diagnosis	1 Not done	6	27.27
	2 Atypical cells	6	27.27
	3 Positive	5	22.73
	4 Negative	5	22.73
	5 Insufficient	0	0.00
Chemotherapy	1 Yes	13	59.09
	2 No	9	40.91
Radiation therapy	1 Yes	4	19.05
	2 No	17	80.95
Smoking status	1 Active	10	45.45
	2 Passive	0	0.00
	3 No	1	4.55
	4 Unknown	9	40.91
	5 Ex-smoker	2	9.09
Surgical treatment	Yes	0	0.00
	No	22	100.00

Survival data

The data showed an overall median survival of 6.4 months (interval = 11). The univariate analysis results for chemotherapy, among all variables, showed a 62% relative reduction in risk of death (hazard ratio, 0.38, P < 0.094), and for radiation therapy, a 54% relative reduction in risk of death (hazard ratio 0.46, P < 0.361). The multivariate analysis results for chemotherapy showed a 77% relative reduction in risk of death (hazard ratio 0.23, P < 0.054), and for radiation therapy, a 65% relative reduction in risk of death (hazard ratio 0.35, P < 0.272) (Table 2).

**Table 2:** Univariate and multivariate analysis for overall survival in patients treated for SCLC SCLC: small cell lung cancer

Variable	Univariate analysis		Multivariate analysis	
	Hazard ratio	P-value	Hazard ratio	P-value
Gender	0.1873913	0.157	0.5664598	0.586
Age	0.9471779	0.382	0.9999046	0.998
Nationality	0.226725	0.144	0.87809	0.830
Chemotherapy	0.2346458	0.054	0.3829413	0.094
Radiation therapy	0.3481099	0.272	0.457047	0.322

## Discussion

The data for patients treated for SCLC in our study showed that the mean age of diagnosis at KAUH, Jeddah, Saudi Arabia, was 62 years, lower than that reported in a study done in Mainz University Hospital, Mainz, Germany. In that study, the mean age of 109 patients newly diagnosed with SCLC was 68 ± 9.1 years [7]. The difference in results could be due to the lack of knowledge about the effects of smoking in Saudi Arabia and to the widespread occurrence of second-hand smoke, leading to earlier exposure to smoking.

Our study also showed a much higher incidence of SCLC among males (90.91%) than among females. Since SCLC is highly associated with smoking, the difference between the sexes could be because it is culturally unacceptable for women to smoke, making it less likely that they will do so. Nevertheless, smoking is increasing among women in Saudi Arabia, which could lead to an increase in the incidence of SCLC. A study that used the Surveillance, Epidemiology, and End Results cancer incidence public database to collect data for 60,045 patients with SCLC from 13 geographic samples, representing approximately 14% of the total US population from 1973 to 2002, initially showed a strong male predominance among patients in 1973 (72.37%). However, this percentage steadily decreased over 20 years until 2002, when the male-to-female ratio reached 1:1 in patients with SCLC [8].

Among the patients in our study, 59% received chemotherapy and had a 62% relative reduction in risk of death (hazard ratio 0.38, P < 0.094), 19% received radiation therapy and had a 55% relative reduction in risk of death, and none underwent surgery. These results could reflect the late detection of SCLC and rapid disease progression. However, the management of SCLC is still a significant challenge for oncologists. Systemic treatment for patients with SCLC has not undergone any remarkable changes in the past several decades, and efforts to improve the outcomes of first-line treatment have all failed to this point. As a result, the five-year survival rate remains dismal at <7% overall, and most patients survive for only one year or less following diagnosis. Unlike the case for NSCLC, in which major advances have been made by using targeted therapies, there are still no approved targeted drugs for SCLC. The lack of real progress in SCLC in recent years emphasizes the importance of developing new treatment strategies [8].

Important barriers to progress in the diagnosis and treatment of SCLC include (1) lack of early detection or appropriate cost-effective screening, (2) limited tumor tissue for translational research (e.g. molecular profiling of DNA, RNA, and/or protein alterations) as a result of the use of small diagnostic biopsies and the rare use of surgical resection in standard treatment, and (3) rapid disease progression with poor understanding of the mechanisms contributing to therapeutic resistance [9].

## Conclusions

In our study, we aimed to assess the outcomes for patients diagnosed with SCLC at KAUH. We observed that more than half of those who received chemotherapy and 19% of those who received radiation therapy showed improvement and a higher survival rate than did patients who did not undergo these treatments. Future efforts to address the major issues for SCLC survivors and to formulate a comprehensive survivorship care plan are required to develop better survival outcomes and to improve the overall quality of life to pretreatment levels.
